# Prognostic nomogram for acute-on-chronic hepatitis B liver failure

**DOI:** 10.18632/oncotarget.21012

**Published:** 2017-09-18

**Authors:** Su Lin, Juan Chen, Mingfang Wang, Lifen Han, Haoyang Zhang, Jing Dong, Dawu Zeng, Jiaji Jiang, Yueyong Zhu

**Affiliations:** ^1^ Liver Research Center, First Affiliated Hospital of Fujian Medical University, Fuzhou, Fujian, China; ^2^ Digestive System Department, Fujian Provincial Hospital, Fuzhou, Fujian, China; ^3^ Department of Infectious Disease, Meng Chao Hepatobiliary Hospital of Fujian Medical University, Fuzhou, Fujian, China; ^4^ Division of Biostatistics, JC School of Public Health and Primary Care, The Chinese University of Hong Kong, Sha Tin, Hongkong, China

**Keywords:** prognosis, survival, liver to abdominal area ratio (LAAR), model for end-stage liver disease (MELD) score, age

## Abstract

**Background & Aims:**

To establish an effective prognostic nomogram for acute-on-chronic hepatitis B liver failure (ACHBLF).

**Materials and Methods:**

The nomogram was based on clinical data of 203 ACHBLF patients who admitted to the First Affiliated Hospital of Fujian Medical University from 2009 to 2014. The area under the receiver-operating characteristic curve (AUC) and calibration curve were carried out to verify the predictive accuracy ability of the nomogram. The result was validated in internal and external validation cohorts. Kaplan-Meier survival curve was used in survival analysis.

**Results:**

We developed a new prognostic nomogram to predict 3-month mortality based on risk factors selected by multivariate analysis. This nomogram consisted three independent factors: age, liver to abdominal area ratio (LAAR) and model for end-stage liver disease (MELD) score. The AUC of this nomogram for survival prediction was 0.877 (95% CI 0.831–0.923), which was higher than that of MELD score, MELD-Na and Child-Turcotte-Pugh (CTP). Good agreement of calibration plot for the probability of survival at 3-month was shown between the prediction by nomogram and actual observation. These results were supported by internal and external validation studies.

**Conclusions:**

The ACHBLF nomogram could predict the short-term survival for ACHBLF patients.

## INTRODUCTION

Acute-on-chronic liver failure (ACLF) is characterized by a precipitating event in patients with underlying chronic liver disease, leading to acute deterioration of liver function and often ending in multi-organ system failure [1]. About 650,000 people worldwide die from liver failure, cirrhosis, and hepatocellular carcinoma, which are caused by hepatitis B virus (HBV) infection each year [2]. HBV has been the major cause of ACLF in the developing countries in Asia. Up to now, the most effective treatment method of ACLF is liver transplantation (LT), but the shortage of liver donors prohibits the widely application of LT. Therefore, careful selection of patients is important for efficient organ allocation. Early identification of patients with poor prognosis can reduce mortality of this disease [3]. Thus, a model that can predict short-term mortality in ACLF patients is necessary for clinicians.

There are many prognostic models for ACLF, such as Child-Turcotte-Pugh(CTP), the model for end-stage liver disease (MELD) score, MELD-Na, sequential organ failure assessment. These prognostic models are based on clinical symptoms and biochemical parameters, not volume of the liver. The majority of acute-on-chronic hepatitis B liver failure (ACHBLF) had underlying liver cirrhosis, which could lead to the changes of liver morphology. Imaging evaluation is an important part of the evaluation of liver function. Saygili et al. [4] found that computed tomography (CT) could assess severity of liver cirrhosis. It was reported that CT-derived liver volume could be used as a prognostic factor for acute liver failure [5, 6]. In those studies, the liver volume was measured by manual measurement or semiautomated measurement. Semiautomated volumetry was convenient but required some special software, which restricted its use. Manual volumetry was time-consuming [7], and also affected by the evaluator’s experience. In contrast, the liver to abdominal area ratio (LAAR) on cross-sectional imaging, which is simply based on CT, could accurately predict mortality in end-stage liver disease [8]. We found that lower LAAR was related to better prognosis in alcoholic ACLF [9]. Inspired by this idea, the aim of the present study was to explore that whether LAAR alone or LAAR combining with currently prognostic methods such as MELD score would provide a more precise and powerful predicting ability on ACHBLF patients. Nomograms have been acknowledged to be accurate in prognostic predictions and been applied in area of cancer researches [10, 11]. So, this study would establish a prognostic nomogram for ACHBLF based on LAAR and MELD score.

### Ethics

The study protocol has been approved by the Institutional Ethics Committee of the First Affiliated Hospital of Fujian Medical University and Meng Chao Hepatobiliary Hospital of Fujian Medical University, and were in compliance with the Declaration of Helsinki.

## MATERIALS AND METHODS

### Patients and treatment

We retrospectively reviewed data on patients who were diagnosed with ACLF, at the First Af?liated Hospital of Fujian Medical University between January 2009 and December 2014 and Meng Chao Hepatobiliary Hospital of Fujian Medical University between January 2015 and December 2015. The diagnosis of ACLF was fulfilled under the guideline of the Asian Pacific Association for the Study of the Liver (2014): the development of jaundice (total serum bilirubin [TBIL] ≥ 5 mg/dl) and coagulopathy (international normalized ration[INR] ≥ 1.5 or prothrombin activity ≤ 40%) was complicated within 4 weeks due to ascites and/or encephalopathy in patients with previously diagnosed or undiagnosed chronic liver disease [12]. We excluded patients with acute liver failure, chronic liver failure, alcoholic liver disease, fatty liver, human immunodeficiency virus, hepatocellular carcinoma, hemolytic jaundice, obstructive jaundice, hematologic neoplasms and coinfection with hepatitis A, C, D, or E viruses. Non-contrast CT was performed in every new diagnosed ACLF, and regular clinical and biochemical data were recorded during follow-up until the death or transplantation.

All quali?ed patients at the First Af?liated Hospital of Fujian Medical University were randomly assigned at a 2:1 ratio into the training cohort to develop the prognostic nomogram and the internal validation cohort to validate the established predictive models. All quali?ed patients from Meng Chao Hepatobiliary Hospital of Fujian Medical University served as the external validation cohort. The primary outcome was death or LT. All patients were classified into two groups: survival group and non-survival group who died or received LT within 3 months.

All the patients received supportive measures, including resting, albumin, nutritional supporting treatment, maintaining of electrolyte balance, antibiotics for infection, terlipressin for hepatorenal syndrome and lactulose for hepatic encephalopathy. All patents got antiviral therapy such as entecavir when they were initially diagnosed the ACHBLF.

### Data collection

The clinical and laboratory data were collected on the day of admission, including the presence of infection or hepatic encephalopathy (HE), upper gastrointestinal bleeding (UGIB), TBIL, albumin (ALB), serum sodium (Na), serum creatinine (Cr), INR, alanine aminotransferase (ALT), aspartate transaminase(AST), hepatitis B surface antigen(HBsAg) levels and hepatitis B virus-deoxyribonucleic acid (HBV DNA) levels.

Infection was diagnosed based on the following criteria [13]: (a) spontaneous bacteremia: positive blood cultures without a source of infection; (b) spontaneous bacterial peritonitis: ascitic fluid polymorphonuclear cells > 250/μL; (c) lower respiratory tract infections: new pulmonary infiltrate in the presence of: (i) at least one respiratory symptom (cough, sputum production, dyspnea, pleuritic pain) with (ii) at least one finding on auscultation (rales or crepitation) or one sign of infection (core body temperature > 38°C or less than 36°C, shivering or leucocyte count > 10,000/mm^3^ or < 4,000/mm^3^) in the absence of antibiotics; (d) Clostridium difficile Infection: diarrhea with a positive C. difficile assay; (e) bacterial entero-colitis: diarrhea or dysentery with a positive stool culture for Salmonella, Shigella, Yersinia, Campylobacter, or pathogenic E. coli; (f) soft-tissue/skin Infection: fever with cellulitis; (g) urinary tract infection: urine white blood cell > 15/high power field with either positive urine gram stain or culture; (h) intra-abdominal infections: diverticulitis, appendicitis, cholangitis etc; (i) other infections not covered above, and (j) fungal infections as a separate category.

### Follow-up

All patients were followed-up for at least 3 months after diagnosis of ACHBLF. Laboratory tests were monitored every 3–5 days in the hospital and then every 1–2 weeks when patients were discharged. The primary outcome was death or LT. The exact time of death of patients was collected through medical records, telephone follow-up and the household registration system queries.

### Calculation of the MELD and MELD-Na

The MELD score was calculated according to the modi?ed Malinchoc [14] formula: R = 9.57 × log e (Cr [mg/dl]) + 3.78 × log e(bilirubin [mg/dl]) + 11.2 × loge (INR) + 6.43 × (etiology: 0 if cholestatic or alcoholic, otherwise 1).

MELD-Na = MELD + 1.59 × (135 - Na), with maximum and minimum Na values of 135 and 120 mEq/L, respectively [15].

### Measurement of LAAR

**Figure 1:**
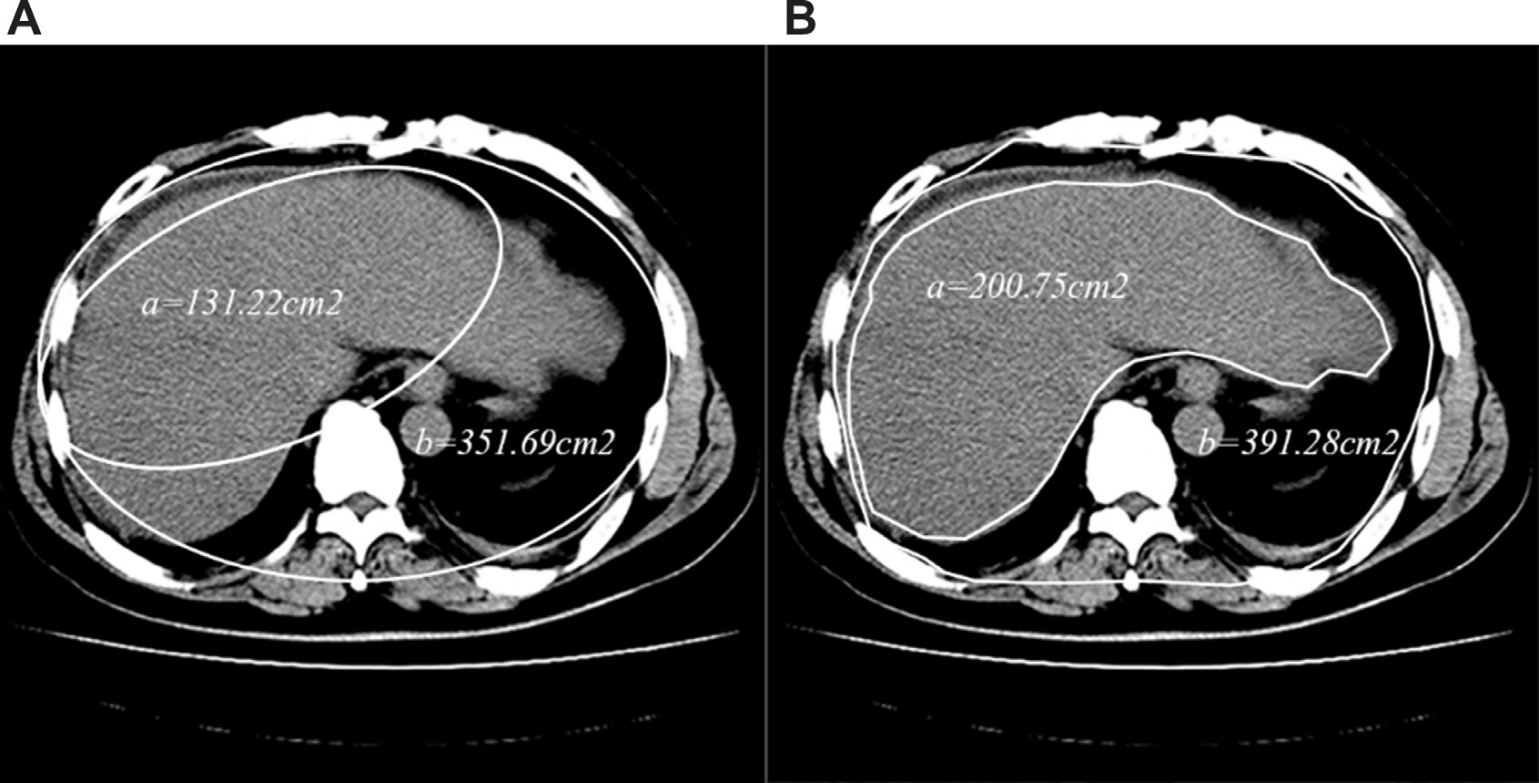
The difference between LAAR (**A**) and developed-LAAR (**B**) calculation schematic diagram. (A) The liver or abdominal area was calculated by drawing a ‘best-fit’ ellipsoid instead of maximum liver or abdominal area, the area was got by calculating the ellipsoid area. (B) The liver or abdominal area was measured by tracing the edge of the liver or the abdomen with a cursor, and then the area would be calculated by the computer automatically.

The whole LAAR of the patients were measured by using the CT ?lms and the method described by Cross et al. [8]. The plain scans were performed on a 320-slice CT scanner (Aquilion One, Toshiba Medical Systems, Otawara, Japan) within 3 days after admission. According to Cross [8], LAAR = liver area (cm^2^)/abdominal area (cm^2^) ×100. The area was calculated by drawing an ellipse which approximates liver area or abdominal area (Figure 1A). We improved the calculation method of area to make it more precise. The liver and abdominal area, which were visualized with plots when the maximum liver area of slice was selected and traced with a cursor, would be calculated by computer automatically (Figure 1B). LAAR was measured by two doctors. The means of LAAR from two different observers were used in final analysis.

### Statistical analysis

All statistical analyses were carried out using SPSS version 13 and R 3.2.2 (http://www.r-project.org/). Baseline comparisons between the two cohorts were performed using student’s *t*-test or Mann-Whitney test for continuous variables where appropriate and chi-square tests for categorical variables. Univariate and multivariable Cox proportional hazard analysis were used to recognize independent prognostic factors. Independent prognostic factors were identified through stepwise selection in a multivariable Cox proportional hazard analysis. Nomogram was established based on the results of multivariable analysis [16]. The discriminative ability of the nomogram was measured by area under the receiver-operating characteristic (ROC) curve(AUC) and calibration curve by comparing the predicted nomogram to actual observed Kaplan-Meier estimates of survival probability. ROC curve was drawn to determine the optimal threshold of nomogram. Kaplan-Meier survival curve was used in survival analysis. A *P*-value < 0.05 was considered statistically signi?cant.

## RESULTS

### The baseline characteristics of the patients

**Figure 2:**
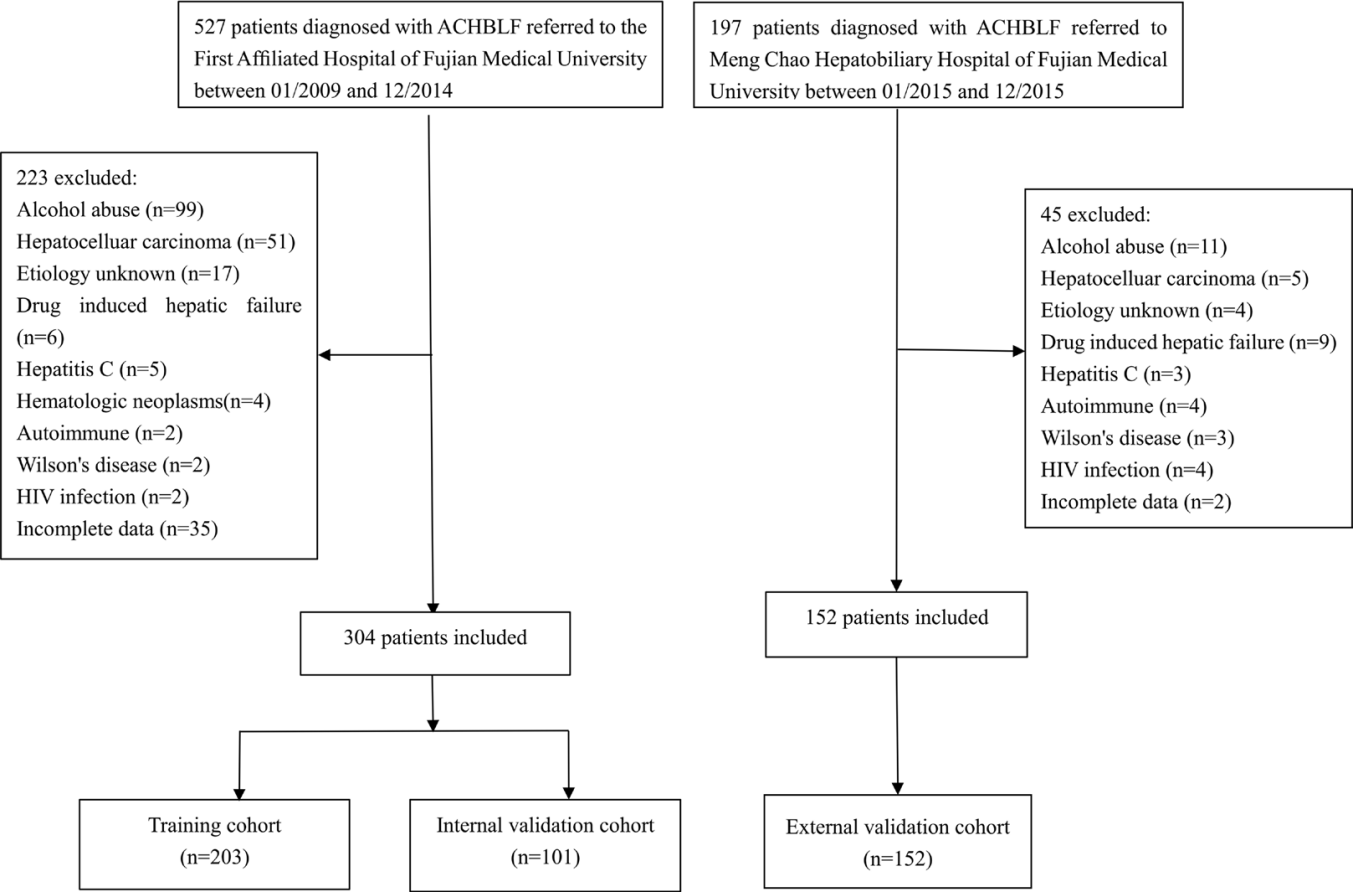
Flow chart of patient selection

From January 2009 to December 2014, 527 patients were diagnosed with ACLF at the First Af?liated Hospital of Fujian Medical University. A total of 304 patients were included in this study after excluding 223 patients (Figure 2). Those patients were randomly assigned into a training cohort (*n* = 203) and an internal validation cohort (*n* = 101). At a median of 494 days (range 2–2454 days) of follow-up, there were 128 patients died and 3 patients underwent LT. The baseline characteristics between the training cohort and the internal validation cohort were not signi?cantly different (Table 1). In the training cohort, the median age was 44 years old, 168 (82.8%) were male, of which 42.4% patients (*n* = 86) died at the end of the follow-up. In the internal validation cohort, the median age also was 44 years old, 85 (84.2%) were male, of which 44.6% patients (*n* = 45) died at the end of the follow-up. The most common complication of ACHBLF was ascites.

**Table 1:** Baseline characteristics of the training and validation sets

Variable	Training (*n =* 203)	Internal validation (*n =* 101)	External validation (*n*=152)	*P* value^*^	*P* value†
Sex, Male *n*, %	168 (82.8)	85 (84.2)	130 (85.5)	0.758	0.527
Age, years	44 (35–54)	44 (35–55)	43 (34–52)	0.932	0.736
Ascites *n*, %	91 (44.8)	53 (52.5)	84 (55.2)	0.208	0.112
HE *n*, %	18 (8.9)	7 (6.9)	21 (13.8)	0.563	0.062
Infection *n*, %	82 (40.4)	35 (34.7)	67 (44.1)	0.333	0.251
UGIB *n*, %	14 (6.9)	8 (7.9)	5 (3.3)	0.745	0.092
Arti?cial liver support system *n*, %	55 (27.1)	32 (31.7)	55 (56.7)	0.404	0.100
TBIL (mmol/L)	303.4 (215.8–427.2)	295.9 (205.1–416.1)	297.8 (226.8–436.3)	0.306	0.583
Sodium (mmol/L)	138.0 (135.0–140.0)	138.0 (135.0–139.2)	137 (134.3–139.7)	0.444	0.181
INR	1.9 (1.6–2.4)	2.0 (1.7–2.5)	2.0 (1.7–2.6)	0.382	0.054
Creatinine (umol/L)	61.7 (54.2–71.9)	60.8 (50.3–71.2)	61.2 (55.8–67.5)	0.257	0.898
Platelet count (109/L)	111 (77–151)	108 (76–139)	114 (81–148.5)	0.711	0.514
MELD score	21.1 (18.5–24.9)	21.3 (18.2–24.7)	21.8 (19.3–25.5)	0.515	0.230
MELD-Na score	21.7 (18.9–26.8)	22.3 (18.3–26.2)	22.7 (19.7–27.5)	0.590	0.186
CTP	10 (9–11)	10 (9–12)	11 (9–12)	0.193	0.115
LAAR	39.9 (35.3–45.4)	38.9 (34.7–44.1)	38.8 (34.7–43.7)	0.306	0.268

Values are expressed as medians (interquartile range).

Abbreviations: HE, hepatic encephalopathy; TBIL, total bilirubin; UGIB, upper gastrointestinal bleeding; INR, international normalized ratio; MELD , model for end-stage liver; CTP, Child-Turcotte-Pugh, LAAR: liver to abdominal area ratio.

^*^Comparison between the training cohort and the internal validation cohort from the First Af?liated Hospital of Fujian Medical University.

†Comparison between the external validation cohort from Meng Chao Hepatobiliary Hospital of Fujian Medical University and all patients in the training and internal validation cohorts from the First Af?liated Hospital of Fujian Medical University.

From January 2015 to December 2015, 197 patients were diagnosed with ACLF at Meng Chao Hepatobiliary Hospital of Fujian Medical University. 152 patients were included to serve as the external validation cohort after excluding 45 patients (Figure 2). In the external validation, the median age was 43 years old, 130(85.5%) were male, of which 33.6% patients (*n* = 51) died at the end of the follow-up. The baseline characteristics between two hospitals were not signi?cantly different (Table 1).

### Prognosis analysis in the training cohort

Univariate analysis showed that age, TBIL, Na, INR, ALB, Cr, ascites, infection, HE, UGIB, LAAR and MELD score differed significantly between sur*vivo*rs and those who died within three months (Table 2). Since TBIL, Cr and INR had linear correlation with MELD score, these three parameters were not taken into multivariate cox regression analysis. On the cox regression analysis, age (hazards ratio [HR] 1.023; 95% con?dence interval [CI] 1.006–1.041), LAAR (HR 0.917; 95% CI 0.884–0.951) and MELD score (HR 1.131; 95% CI 1.092–1.171) were independent risk factors for 3-months mortality.

**Table 2:** Baseline characteristics of survival and non-survival patients in training group

Variables	survival group (*n =* 117)	non-survival group (*n =* 86)	Univariate analysis	Multivariate analysis		
			*P*-value	HR	*P*	95% CI
Age, years	41 (34–49)	48 (39–59)	< 0.001	1.023	0.008	1.006–1.041
Male *n*, %	98 (83.8)	70 (81.4)	0.525			
TBIL(umol/L)	258.4 (197.2–389.1)	331.1 (262.4–473.0)	< 0.001			
ALT(U/L)	332.0 (120.5–826.0)	269.5 (106.5–751.5)	0.690			
AST(U/L)	297.0 (131.0–586.5)	268.5 (106.5–586.5)	0.797			
ALB(g/L)	30.7 (28.5–34.3)	29.8 (27.0–33.1)	0.035			
Cr (umol/L)	61.0 (54.7–68.4)	63.0 (53.7–84.7)	< 0.001			
Platelet count (10^9^/L)	115.0 (81.5–154.0)	107.5 (64.75–150.0)	0.205			
Na (mmol/L)	139.0 (136.0–141.0)	136.0 (132.1–139.0)	< 0.001			
INR	1.7 (1.6–2.0)	2.2 (1.8–3.0)	< 0.001			
HBsAg (IU/ml)	2675.2 (557.3–10515.0)	1555.0 (418.7–7167.3)	0.100			
HBV DNA (log10[IU/ml])	4.5 (3.3–6.0)	4.74 (3.4–6.0)	0.754			
Arti?cial liver support system *n*, %	26 (22.2)	29 (33.7)	0.064			
Ascites *n*, %	38 (32.5)	53 (61.6)	< 0.001			
HE *n*, %	2 (1.7)	16 (18.6)	< 0.001			
Infection *n*, %	34 (29.1)	48 (55.8)	< 0.001			
UGIB *n*, %	4 (3.4)	10 (11.6)	0.010			
LAAR	43.2 (38.9–47.7)	35.8 (32.1–39.5)	< 0.001	0.917	< 0.001	0.884–0.951
MELD	19.9 (17.6–21.9)	24.9 (20.9–29.2)	< 0.001	1.131	< 0.001	1.092–1.171

Abbreviations: TBIL, total bilirubin; ALT, alanine aminotransferase; AST, aspartate transaminase; ALB, albumin; Cr, serum creatinine; Na, serum sodium; INR, international normalized ratio; HBsAg, hepatitis B surface antigen; HBV-DNA, hepatitis B virus -deoxyribonucleic acid; HE, hepatic encephalopathy; UGIB, upper gastrointestinal bleeding; LAAR: liver to abdominal area ratio; MELD, model for end-stage liver disease; HR, hazards ratio; CI, con?dence interval.

### Prognostic Nomogram for survival in training cohort

**Figure 3:**
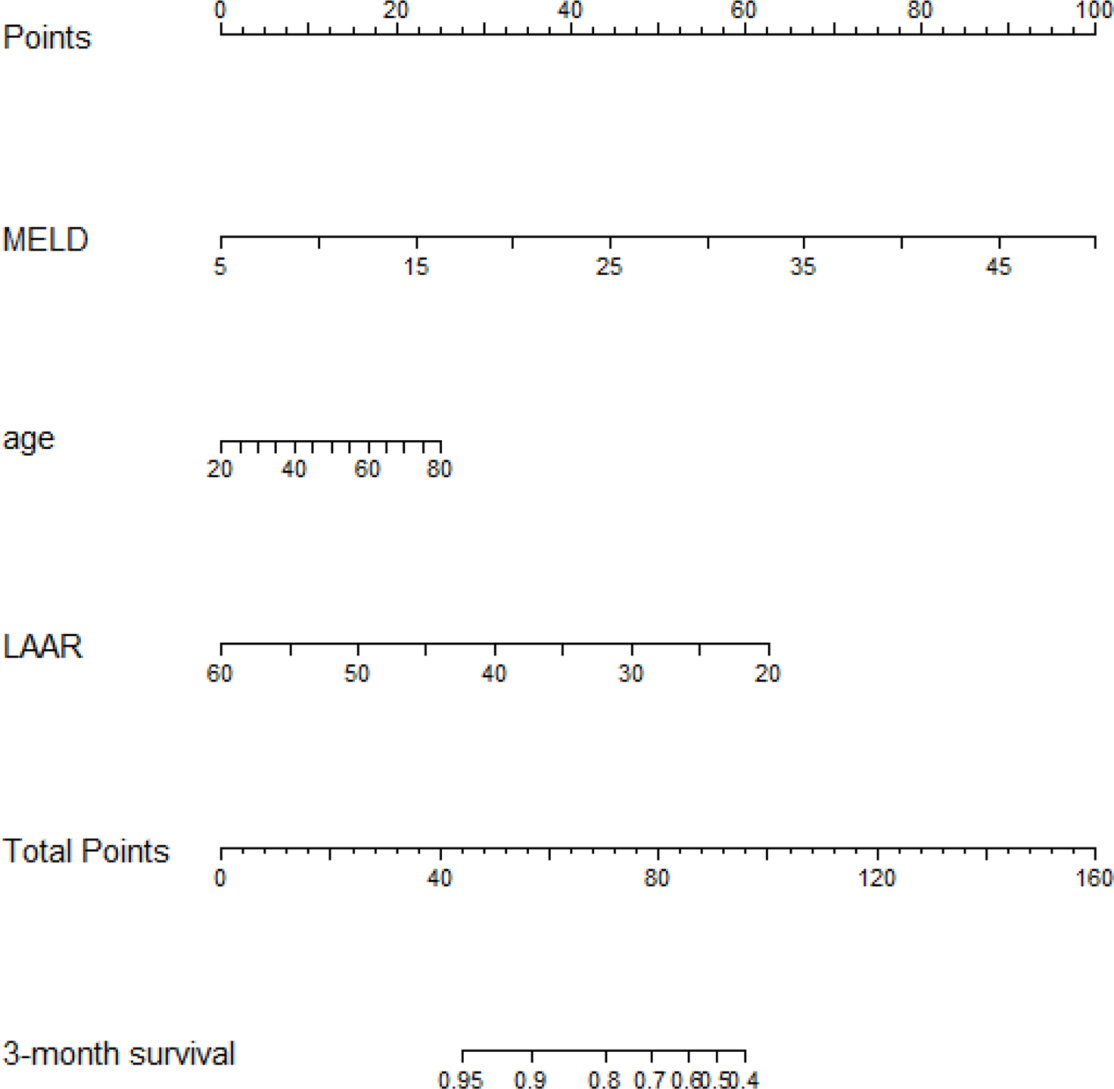
Nomogram to predict overall survival in ACHBLF patients Draw an upward vertical line from each variable axis to the points bar to get points of each variable. Based on the sum of each variable points, draw a downward vertical line from Total Points axis to calculate 3-month overall survival.

**Figure 4:**
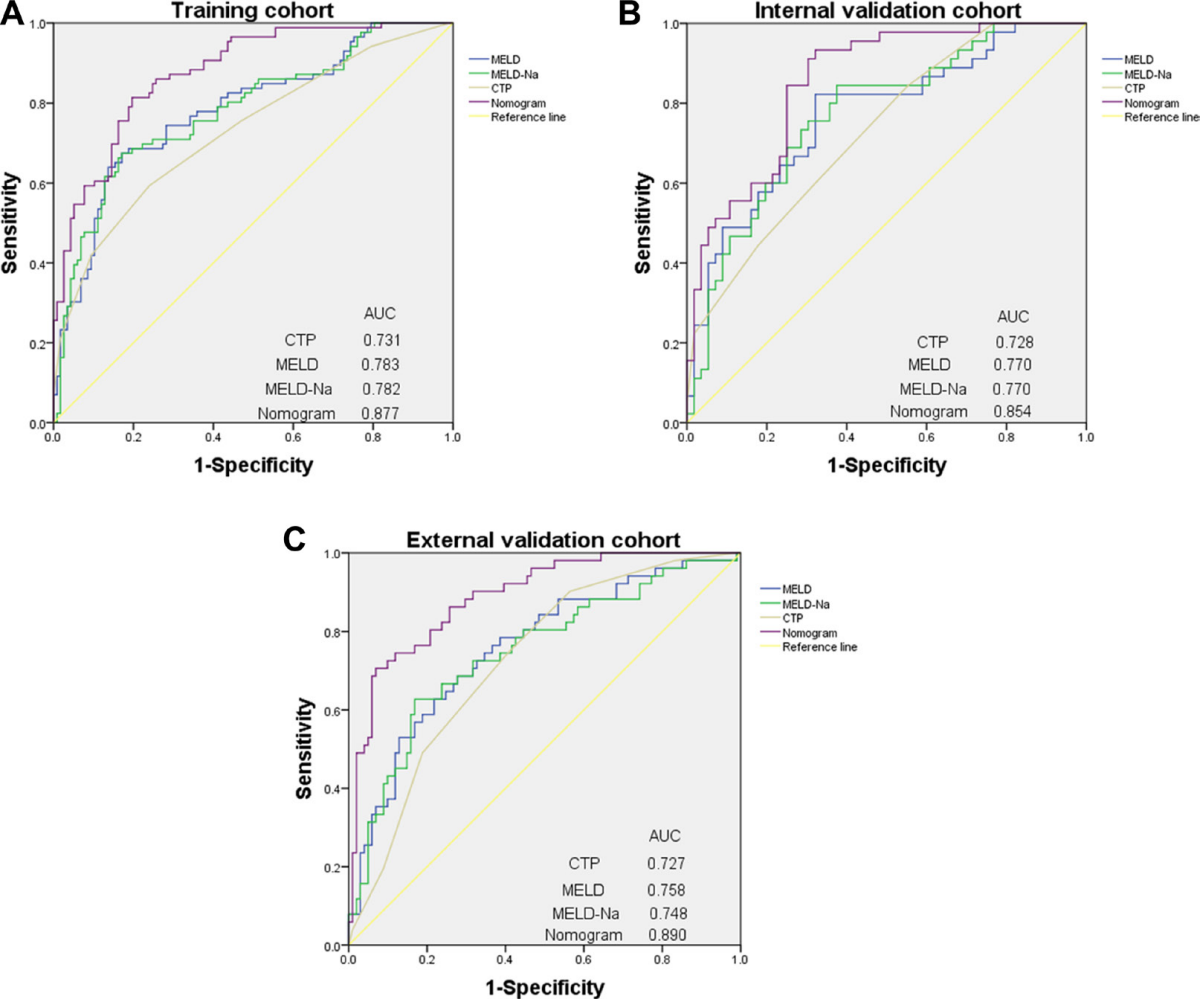
ROC curve of nomogram and other models to predict morbidity of patients with ACHBLF (**A**) ROC curve in training cohort. (**B**) ROC curve in internal validation cohort. (**C**) ROC curve in external validation cohort.

**Figure 5:**
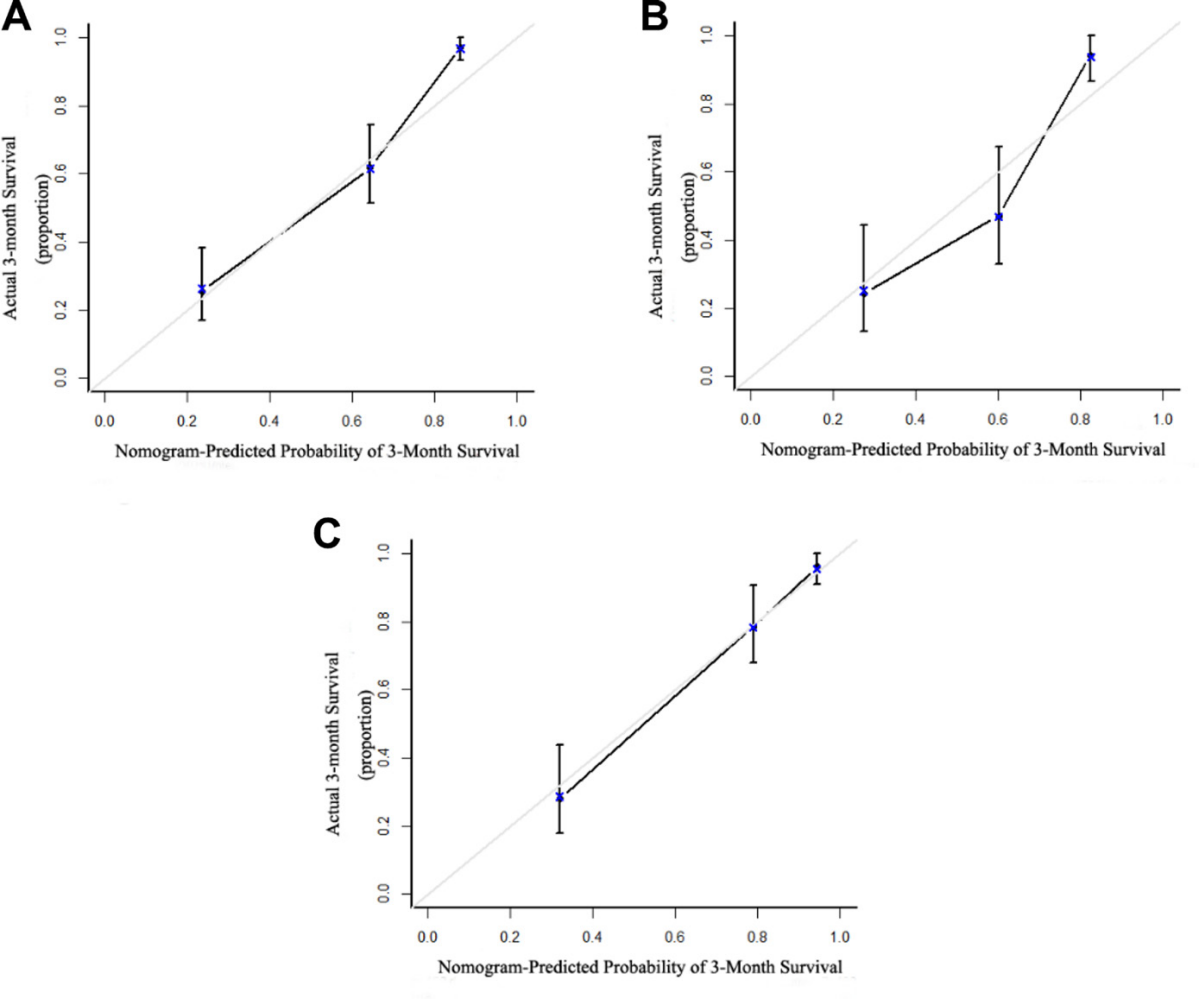
The calibration curve for predicting patient survival (**A**) Calibration curves for predicting 3-month overall survival rate in the training cohort. (**B**) Calibration curves for predicting 3-month overall survival rate in the internal validation cohort. (**C**) Calibration curves for predicting 3-month overall survival rate in the external validation cohort. X axis is the nomogram-predicted probability of overall survival; y axis is the actual overall survival in the calibration curves.

The independent risk factors for prognosis of ACHBLF, age, LAAR and MELD score were incorporated into the nomogram (Figure 3). The AUC of the nomogram for survival prediction was 0.877 (95% CI 0.831–0.923), while the AUC was 0.783 (95% CI 0.719–0.848) for MELD score, 0.782 (95% CI 0.717–0.847) for MELD-Na and 0.731 (95% CI 0.660–0.801) respectively (Figure 4A). The difference was statistically signi?cant between nomogram and MELD score (*P* = 0.001), between nomogram and MELD-Na (*P* = 0.001) or between nomogram and CTP (*P* < 0.001). The calibration plot for the probability of overall survival at 3-month demonstrated good agreement between the prediction by nomogram and actual observation (Figure 5A).

### Nomogram for overall survival prediction in internal validation cohort

Using 3-month mortality as the end point, the AUC was 0.854 (95% CI 0.782–0.926) for the nomogram, 0.770 (95% CI 0.677–0.863) for the MELD score, 0.770 (95% CI 0.678–0.862) for MELD-Na, 0.728 (95% CI0.632–0.824) for CTP, respectively. The difference was statistically signi?cant between nomogram and MELD score (*P* = 0.027), between nomogram and MELD-Na (*P* = 0.028) or between nomogram and CTP (*P* = 0.003) (Figure 4B). The calibration plot showed good agreement between prediction and observation in the probability of 3-month survival (Figure 5B).

### Nomogram for overall survival prediction in external validation cohort

Using 3-month mortality as the end point, the AUC was 0.890 (95% CI 0.836–0.943) for the nomogram, 0.758 (95% CI 0.676–0.840) for the MELD score, 0.748(95% CI 0.663–0.833) for MELD-Na, 0.727 (95% CI 0.645–0.803) for CTP, respectively. The difference was statistically signi?cant between nomogram and MELD score (*P* < 0.001), between nomogram and MELD-Na (*P* < 0.001) or between nomogram and CTP (*P* <0.001) (Figure 4C). The calibration plot showed good agreement between prediction and observation in the probability of 3-month survival (Figure 5C).

### Performance of the Nomogram in Stratifying Risk of Patients

**Figure 6:**
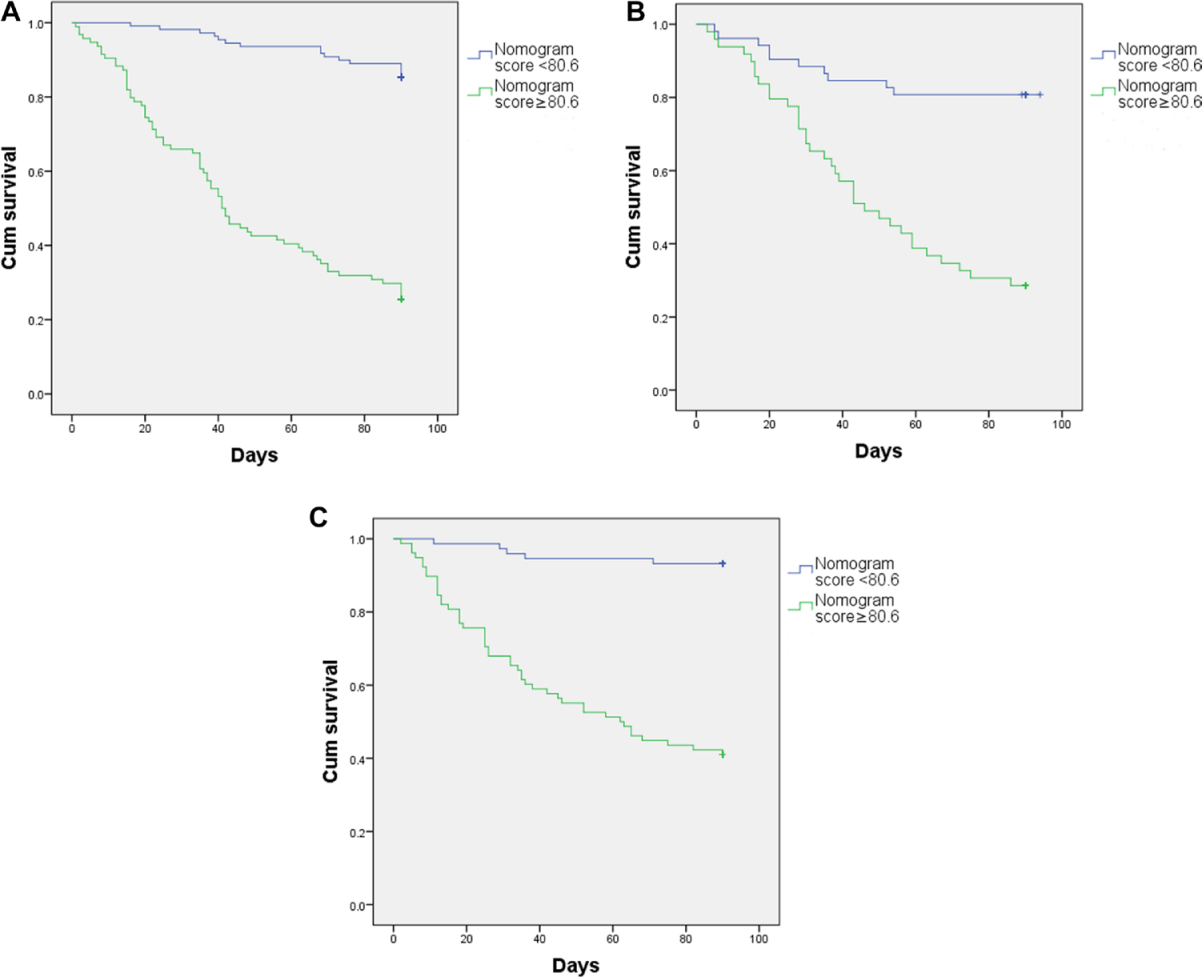
Kaplan-Meier survival curve (**A**) training cohort. (**B**) internal validation cohort. (**C**) external validation cohort.

Kaplan-Meier survival curve was used for survival analysis. The cutoff point about the total score of nomogram was 80.6 in the training cohort. The Kaplan-Meier survival curves with the optimal cutoff was shown in Figure 6A. The 3-month survival rates were 85.32% in the low nomogram score group (nomogram score < 80.6) and 25.53% in the high nomogram score group (nomogram score ≥ 80.6), respectively. The 3-month survival rate was significantly decreased when the total score of nomogram > 80.6. Log-rank survival analysis showed a signi?cant difference (*P* < 0.001).

After applying the cutoff value to group patients in internal cohort (Figure 6B) and external cohort (Figure 6C), Log-rank survival analysis showed a signi?cant difference (*P* < 0.001).

### Usage of nomogram

For example, if a patient gets the MELD score = 20, drawing an upward vertical line from MELD variable axis to the point bar to get point of MELD variable is 33. If LAAR of this patient is 50, the point corresponding to LAAR variable is 16. If the age of a patient is 70, the corresponding point is 21. The total points equal to 70 (33 + 16 + 21). So, 3-month overall survival is 80% when we draw a straight line from Total Points axis to 3-month overall survival axis (Figure 3).

## DISCUSSION

ACLF is a life-threatening event with high mortality. In our study, three-month mortality of ACHBLF was 40.0%, which was similar to other literature data [17–19]. We developed ACHBLF nomogram which could predict the short-term prognosis of liver failure. In the nomogram, the AUC for survival prediction were 0.877, 0.854 and 0.890 in the training, internal validation cohorts and external validation cohorts, respectively, and were significantly higher than those of MELD scores, MELD-Na and CTP, suggesting a favorable prediction ability. Survival was significantly decreased when the total score of nomogram > 80.6.

Death of a large number of hepatocyte plays a crucial role in the development of liver failure. The liver volume could partially re?ect the quantity liver cells. Liver volume has been reported to have a significant correlation with the liver function [4, 20] and be closely related to the prognosis of acute liver failure [5, 6, 21]. However, the method of liver volume calculation is complicated, which limits its use. Therefore, Cross et al. [8] developed a simple, convenient and easy parameter, LAAR, to replace CT-derived liver volume. The LAAR has already been demonstrated to have an excellent ability in predicting the prognosis of liver cirrhotic patients [8]. In our study, lower LAAR also showed a significant relationship with poor outcome of ACHBLF.

In COX regression analysis, LAAR and MELD scores were independent prognostic factors for ACHBLF. MELD score was proposed by Kamath, composed of Cr, TBIL, INR and etiology of liver failure. MELD score could reflect multiple organ functions, such as liver, kidney and blood coagulation function, which closely related to liver failure. Many researches [22, 23] indicated that MELD score had good predictive capacity about ACLF prognosis. However, MELD score had many shortcomings, such as the variation of INR by different laboratory methodologies; Cr would be influenced by age, gender and body mass index of the patients [24]. The validation of MELD score in predicting outcome of ACHBLF was still controversial. Angermayr et al. [25] found that the efficiency of MELD score in predicting 1-year survival depends the etiology of cirrhosis: viral cirrhosis patients with MELD ≥ 16 would have significantly lower survival rates than alcoholic patients with same MELD scores. The ACHBLF nomogram contained MELD score and LAAR, reflecting both liver function and liver volume, which could complement each other perfectly. The AUC of ACHBLF nomogram were 0.877, which was higher than that of MELD score (AUC 0.783, *P* = 0.001), indicating that this new model had a better predictive power.

In our research, age was an independent prognostic factor for ACHBLF, which was consistent with the previous studies [22, 26]. Xie et al. [27] found that the mortality of HBV related liver failure increased markedly with increasing age ≥ 35 years in males and ≥ 55 years in females. In patients with chronic liver disease, liver function deteriorates with age and the regenerative capacity of the liver declines [28]. The older patients are more easily combined with complications because of low immunity.

After all, the ACHBLF nomogram consists of 3 components: age, LAAR and MELD score. This model predicts the prognosis of liver failure from several aspects: imaging and biochemistry. It is a convenient and clearly straightforward method to get probability.

There are several limitations to this study. We only included HBV-induced ACLF. The common etiologies of ACLF were alcohol, hepatitis B, hepatitis C, and non-alcoholic fatty liver disease [12]. In Asia, chronic hepatitis B accounts for 80% population developing ACLF [29], but alcoholic liver disease only accounts for 13% [30]. The LAAR in alcoholic ACLF were relatively larger than ACHBLF, and the larger LAAR in alcoholic patients were associated with higher mortality in our previous study [9]. This conflict results made it impossible to combine two different cause of ACLF into one predictive model. Hepatitis C, autoimmune hepatitis and Wilson’s disease were less common causes of ACLF in our department. The data were not enough for analysis. So, we were not able to incorporate cases except for HBV into new model due to the insufficient sample size and different physiopathological status. Although this nomogram was developed based on ACHBLF, it did not contain any specific serological markers of hepatitis B virus. The validation of this nomogram in other causes of liver failure is worth of further investigation.

## CONCLUSIONS

In conclusion, we have developed ACHBLF nomogram that could predict short-term prognosis of ACHBLF patients.

## Abbreviations

ACLF: acute-on-chronic liver failure
HBV: hepatitis B virus
LT: liver transplantation
CTP: Child-Turcotte-Pugh
MELD: model for end-stage liver disease
ACHBLF: acute-on-chronic hepatitis B liver failure
CT: computed tomography
LAAR: liver to abdominal area ratio
TBIL: total bilirubin
INR: international normalized ratio
HE: hepatic encephalopathy
UGIB: upper gastrointestinal bleeding
ALB: albumin
Na: serum sodium
Cr: serum creatinine
ALT: alanine aminotransferase
AST: aspartate transaminase
HBsAg: hepatitis B surface antigen
HBV-DNA: hepatitis B virus-deoxyribonucleic acid
AUC: area under the receiver-operating characteristic curve
ROC: receiver-operating characteristic
HR: hazards ratio
CI: con?dence interval
